# Metabolic compensation constrains the temperature dependence of gross primary production

**DOI:** 10.1111/ele.12820

**Published:** 2017-08-29

**Authors:** Daniel Padfield, Chris Lowe, Angus Buckling, Richard Ffrench‐Constant, Student Research Team, Simon Jennings, Felicity Shelley, Jón S. Ólafsson, Gabriel Yvon‐Durocher

**Affiliations:** ^1^ Environment and Sustainability Institute University of Exeter Penryn Cornwall TR10 9EZ UK; ^2^ Centre for Ecology and Conservation College of Life and Environmental Sciences University of Exeter Penryn Cornwall TR10 9FE UK; ^3^ Centre for Environment Fisheries and Aquaculture Science Lowestoft NR33 0HT UK; ^4^ School of Environmental Sciences Norwich Research Park University of East Anglia Norwich NR4 7TJ UK; ^5^ International Council for the Exploration of the Sea H. C. Andersens Boulevard 44‐46 1553 Copenhagen V Denmark; ^6^ School of Biological and Chemical Sciences Queen Mary University of London London E1 4NS UK; ^7^ Marine and Freshwater Research Institute Árleyni 22 112 Reykjavik Iceland

**Keywords:** Global warming, gross primary production, metabolic theory

## Abstract

Gross primary production (GPP) is the largest flux in the carbon cycle, yet its response to global warming is highly uncertain. The temperature dependence of GPP is directly linked to photosynthetic physiology, but the response of GPP to warming over longer timescales could also be shaped by ecological and evolutionary processes that drive variation in community structure and functional trait distributions. Here, we show that selection on photosynthetic traits within and across taxa dampens the effects of temperature on GPP across a catchment of geothermally heated streams. Autotrophs from cold streams had higher photosynthetic rates and after accounting for differences in biomass among sites, biomass‐specific GPP was independent of temperature in spite of a 20 °C thermal gradient. Our results suggest that temperature compensation of photosynthetic rates constrains the long‐term temperature dependence of GPP, and highlights the importance of considering physiological, ecological and evolutionary mechanisms when predicting how ecosystem‐level processes respond to warming.

## Introduction

The carbon cycle is fundamentally metabolic (Falkowski *et al*. 2000). At the ecosystem‐level, gross primary production (GPP) represents the total amount of CO_2_ fixed by photosynthesis into organic carbon and is the largest flux in the global carbon cycle (Beer *et al*. 2010), transferring CO_2_ from the atmosphere to the biosphere, fuelling food webs and biological production (Field 1998). Understanding the mechanisms that shape how temperature influences rates of GPP across spatial, temporal and organisational scales is therefore an essential prerequisite to forecasting feedbacks between global warming and the carbon cycle.

Temperature can dictate rates of GPP over short timescales through its effects on photosynthetic physiology (Medlyn *et al*. 2002; Allen *et al*. 2005; Galmes *et al*. 2015). However, it is clear that over longer timescales (e.g. decades of gradual warming) ecological and evolutionary processes that mediate temperature‐induced changes in biomass, community composition and local adaptation of metabolic traits could feed back to influence the emergent effects of warming on ecosystem properties (Allen *et al*. 2005; Enquist *et al*. 2007; Michaletz *et al*. 2014; Cross *et al*. 2015). Indeed a recent analysis demonstrated that most of the variation in terrestrial primary production along a latitudinal temperature gradient could be explained by changes in biomass; after controlling for variation in biomass, rates were independent of temperature (Michaletz *et al*. 2014). Such temperature invariance in biomass‐specific rates of primary production is counterintuitive considering the well‐known exponential effects of temperature on the biochemistry of metabolism (Gillooly *et al*. 2001). Furthermore, it implies that selection on photosynthetic traits that compensate for the effects of temperature on physiological rates could play a fundamental role in mediating the effects of temperature on rates of primary production in the long‐term (Kerkhoff *et al*. 2005; Enquist *et al*. 2007).

Here we investigate how rates of ecosystem‐level GPP are influenced by direct effects of temperature on the kinetics of photosynthesis, indirect effects of temperature‐driven selection on photosynthetic traits, and changes in community biomass. We do so by extending the general model for ecosystem metabolism from metabolic scaling theory (Enquist *et al*. 2003, 2007; Allen *et al*. 2005; Kerkhoff *et al*. 2005; Michaletz *et al*. 2014) to account for changes in key traits that influence the thermal response of individual metabolism, as well as potential temperature effects on ecosystem biomass pools. We then test our model's predictions against empirical data collected from a catchment of naturally warmed Icelandic geothermal streams spanning a gradient of 20 °C.

## Theory

Metabolic scaling theory provides a powerful framework for understanding how temperature affects GPP by linking the photosynthetic rates of an ecosystem's constituent individuals with the size and biomass structure of the community (Enquist *et al*. 2003, 2007; Allen *et al*. 2005; Kerkhoff *et al*. 2005; Yvon‐Durocher & Allen 2012; Michaletz *et al*. 2014).

### The temperature dependence of whole‐organism metabolic rate

**Figure 1:**
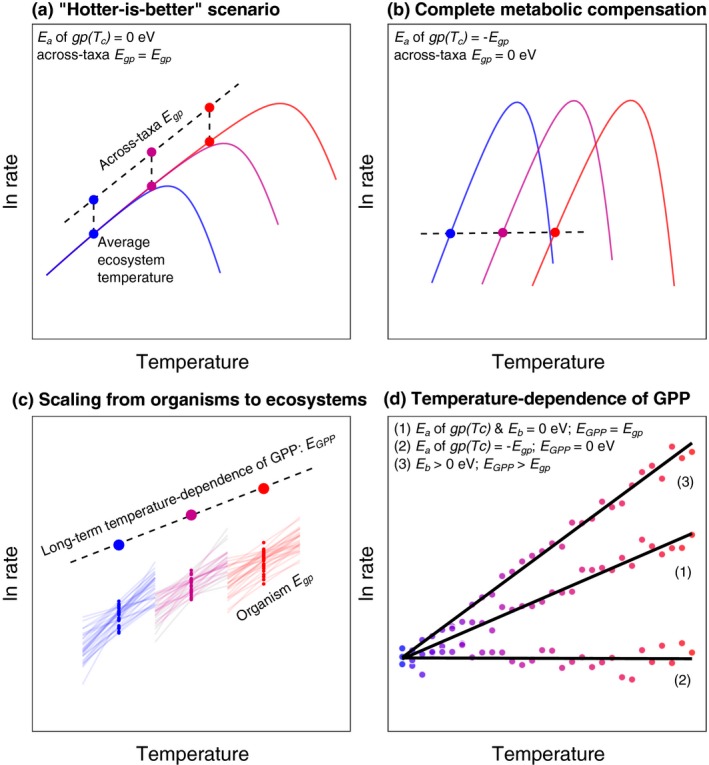
Scaling metabolism from organisms to ecosystems.

The rate of metabolism at the organism‐level, b, responds predictably to temperature, T, increasing exponentially up to an optimum, followed by a more pronounced exponential decline (Fig. 1a). This response can be quantified using a modification of the Sharpe–Schoolfield equation for high‐temperature inactivation (Schoolfield *et al*. 1981):

(1) $$b\left(T\right)=\frac{b\left(T_{c}\right)m^{\alpha }e^{E\left(\frac{1}{kT_{c}}-\frac{1}{kT}\right)}}{1+e^{E_{h}\left(\frac{1}{kT_{h}}-\frac{1}{kT}\right)}}$$

where T is in Kelvin (K), *k* is Boltzmann's constant (8.62 × 10^−5^ eV K^−1^) and E is the activation energy (in eV). $E_{h}$ characterises temperature‐induced inactivation of enzyme kinetics above $T_{h}$, which is the temperature at which half the enzymes are inactivated. In this expression, $b\left(T_{c}\right)$ is the organism‐level rate of metabolism normalised to a reference temperature (e.g. 10 °C), where no low‐ or high‐temperature inactivation occurs and $m^{\alpha }$ is the mass‐dependence of metabolic rate characterised by an exponent α*,* that ranges between ¾ and 1 across multicellular and unicellular autotrophs (Gillooly *et al*. 2001; DeLong *et al*. 2010). Equation (1) yields a maximum metabolic rate at an optimum temperature,

(2) $$T_{opt}=\frac{E_{h}T_{h}}{E_{h}+kT_{h}\ln\left(\frac{E_{h}}{E}-1\right)}$$

The parameters in eqns (1) and (2), which govern the height and shape of the thermal response curve can be considered ‘metabolic traits’ (Padfield *et al*. 2016) and have long been known to reflect adaptation to the prevailing thermal environment (Berry & Bjorkman 1980; Huey & Kingsolver 1989). Equation (1) can be simplified to the Arrhenius equation,

(3) $$b\left(T\right)=b\left(T_{c}\right)m^{\alpha }e^{E\left(\frac{1}{kT_{c}}-\frac{1}{kT}\right)}$$

which captures only the rising part of the thermal response curve. This is applicable when the temperatures organisms experience in the environment are below $T_{opt}$ (Savage *et al*. 2004; Dell *et al*. 2011; Sunday *et al*. 2012). We use this simpler, more tractable model of the temperature‐dependence in the following theory, which attempts to explore the mechanisms driving the emergent temperature‐dependence of ecosystem‐level GPP. At the organism‐level, the size and temperature dependence of gross photosynthesis can be characterised as:

(4) $$gp\left(T\right)=gp\left(T_{c}\right)m^{\alpha }e^{E_{gp}\left(\frac{1}{kT_{c}}-\frac{1}{kT}\right)}$$

where $gp\left(T\right)$ is the rate of gross photosynthesis at temperature T, $gp\left(T_{c}\right)$ is the rate of gross photosynthesis normalised to a reference temperature $T_{c}$ and $E_{gp}$ is the activation energy of gross photosynthesis. Net photosynthesis, np, which is the amount of photosynthate available for allocation to biomass production after accounting for autotroph respiration is given by,

(5) $$\begin{array}{cc} np\left(T\right)= & gp\left(T_{c}\right)m^{\alpha }e^{E_{gp}\left(\frac{1}{kT_{c}}-\frac{1}{kT}\right)}-r\left(T_{c}\right)m^{\alpha }e^{E_{r}\left(\frac{1}{kT_{c}}-\frac{1}{kT}\right)}\\ & =np\left(T_{c}\right)m^{\alpha }e^{E_{np}\left(\frac{1}{kT_{c}}-\frac{1}{kT}\right)} \end{array}$$

where $np\left(T\right)$ is the rate of net photosynthesis at temperature T, $r(T_{c})$ is the rate of autotrophic respiration normalised to a reference temperature, $T_{c}$, and $E_{np}$ and $E_{r}$ are the activation energies of net photosynthesis and autotrophic respiration. Equation (5) implies that the temperature sensitivity of np will not strictly follow a simple Boltzmann–Arrhenius relation. Nevertheless, we can approximate the temperature sensitivity of net photosynthesis using an apparent activation energy, $E_{np}$, with a reasonable degree of accuracy (see supplementary methods for a derivation of $E_{np}$ and Fig. S7 for a test of this approximation).

### Scaling metabolism from organisms to ecosystems

Using eqn (4) and principles from metabolic scaling theory, the rate of gross primary productivity per unit area of an ecosystem, *A*, can be approximated by the sum of the photosynthetic rates of its constituent organisms (see Box 1):

(6) $$GPP_{s}\left(T\right)=GPP\left(T_{c}\right)e^{E_{GPP}\left(\frac{1}{kT_{c}}-\frac{1}{kT}\right)}$$

where $GPP_{s}\left(T\right)$ is the rate of GPP in ecosystem *s*, at temperature *T*, and $GPP\left(T_{c}\right)=\frac{1}{A}\sum_{i=1}^{n}gp_{i}\left(T_{c}\right)m_{i}^{\alpha }$ is the ecosystem‐level metabolic normalisation constant, where *n* is the total number of individual organisms, *i*, which comprise all autotrophs in *s*. In eqn (6), the temperature‐dependence of ecosystem‐level GPP, $E_{GPP}$, is assumed to be equal to that of the average temperature dependence for individual‐level gross photosynthesis, $E_{gp}$, provided that the ecosystem‐level normalisation, $GPP\left(T_{c}\right)$, is independent of temperature (Box 1; Fig. 1d). However, if $gp_{i}\left(T_{c}\right)$ or total autotrophic biomass, $M_{s}=\frac{1}{A}\sum_{i=1}^{n}m_{i}$, exhibit temperature‐dependence, constant for example via acclimation or adaptation acting on $gp_{i}\left(T_{c}\right)$ or covariance between resource availability, temperature and $M_{s}$, then the linear scaling of the activation energy from individuals to ecosystems will no longer hold (e.g. $E_{GPP}\neq E_{gp}$). Thus, ecological processes that influence $M_{s}$ and evolutionary dynamics which shape variation in $gp_{i}\left(T_{c}\right)$ have the potential to play an integral, but as yet underappreciated role in mediating the response of ecosystem metabolism to temperature (Kerkhoff *et al*. 2005; Davidson & Janssens 2006; Enquist *et al*. 2007; Michaletz *et al*. 2014).

Box 1 **Simulating the direct and indirect effects of temperature on gross primary production**

Using metabolic scaling theory (see eqns (1), (2), (3), (4), (5), (6), (7), (8)), we can investigate alternative hypotheses on the effects of temperature‐driven selection and covariance between biomass and temperature on the long‐term temperature‐dependence of gross primary production (GPP). We define the long‐term temperature‐dependence of GPP as that derived across ecosystems that differ in average temperature. To determine the GPP for any given ecosystem, we define an arbitrary number of taxa (e.g. *n *=* *30), assigning each a mass, m, drawn from a normal distribution with a mean of 100 mg and an abundance, N, as $N\propto m^{-3/4}$. The total biomass of each taxon is then calculated as the product, Nm. The rate of gross photosynthesis for each taxon is calculated using eqn (4), with a size scaling exponent of α = ¾ and values for $E_{gp}$ and $gp_{i}\left(T_{c}\right)$ which are drawn from a normal distribution with means of 0.6 eV and 10 μmol O_2_ μg Chl *a* ^−1^ h^−1^ respectively. GPP is then the sum of the gross photosynthetic rates of all organisms comprising the biomass pool at a given temperature (Fig. 1c).

To explore a range of hypotheses for the indirect effects of temperature‐driven selection on the photosynthetic normalisation constant, $E_{a}$, and biomass‐temperature covariance, $E_{b}$, on the long‐term temperature‐dependence of GPP, $E_{GPP}$, we simulated 30 ecosystems each consisting of 30 taxa along a gradient in temperature (10 to 50 °C). In scenario 1, there is no temperature‐driven selection on the photosynthetic normalisation constant, $gp\left(T_{c}\right)$, and biomass is independent of temperature (*E*_*b*_ & *E*_*a*_ = 0 eV; Fig 1a). Under these circumstances the long‐term temperature‐dependence of GPP will be equal to the average temperature‐dependence of organism‐level gross photosynthesis ($E_{GPP}=E_{gp}$; Fig. 1d (1)). In scenario 2, we simulate the effects of complete temperature compensation of organism‐level gross photosynthesis (Fig. 1b), by making the temperature dependence of $gp\left(T_{c}\right)$ equal, but of opposite sign to that of $E_{gp}$ ($E_{a}=-E_{gp}$), with biomass independent of temperature ($E_{b}$ = 0 eV). Under this scenario, long‐term GPP is independent of temperature (Fig. 1d (2)). In scenario (3), we allow biomass to positively covary with temperature ($E_{b}=E_{gp}$), whilst making $gp\left(T_{c}\right)$ temperature‐invariant ($E_{a}$ = 0 eV). In this case, the long‐term temperature dependence of GPP is amplified with respect to that of organism‐level gross photosynthesis ($E_{GPP}>E_{gp}$; Fig. 1d (1)).

These simulations demonstrate how indirect effects of temperature‐driven selection on the photosynthetic normalisation constant and covariance between biomass and temperature can have as large an effect on the emergent temperature‐dependence of ecosystem metabolism as the direct effects of temperature on the kinetics of photosynthesis. In panels a:d blue denotes cold and red warm temperatures.

### Incorporating indirect effects of temperature on ecosystem metabolism

Previous work on aquatic and terrestrial autotrophs has shown that autotrophs can adjust their respiratory and photosynthetic normalisation constants; up‐regulating rates at low temperatures and down‐regulating at high temperature to compensate for the constraints of thermodynamics on enzyme kinetics (Atkin *et al*. 2015; Padfield *et al*. 2016; Reich *et al*. 2016; Scafaro *et al*. 2017). Such changes may be manifest in several ways. First, over relatively short time scales (e.g. within the generation time of an individual) acclimation of cellular physiology (a form of phenotypic plasticity) can result in adjustments to photosynthetic and respiratory capacity that partially compensate for the effects of changes in temperature (Atkin & Tjoelker 2003; Yamori *et al*. 2014). Second, over multiple generations, adaptive evolution driven by natural selection on traits that influence metabolism can also result in temperature compensation of photosynthetic and respiratory capacity. Such evolutionary shifts in metabolic traits have been shown to occur both via rapid micro‐evolutionary responses, resulting in warm‐ or cold‐adapted genotypes of the same species (Padfield *et al*. 2016; Schaum *et al*. 2017) as well as macro‐evolutionary divergence in metabolic traits among different species (Addo‐Bediako *et al*. 2002; Deutsch *et al*. 2008; Sunday *et al*. 2012). Finally, when scaling up to an ecosystem, the distribution of metabolic traits across the constituent individuals will emerge from temperature‐driven selection on trait variation arising both within and among species. When temperature imposes a strong selective force, and variations in temperature are maintained over time scales that span multiple generations (e.g. over spatial thermal gradients or due to global warming), we expect temperature‐driven changes in $gp_{i}\left(T_{c}\right)$ along thermal gradients to reflect selection on trait variation within and among taxa that has arisen via adaptive evolution. In the absence of an explicit first‐principles derivation, we can approximate the effects of temperature‐driven selection on $gp_{i}\left(T_{c}\right)$ as

(7) $$gp_{i}\left(T_{c}\right)\approx e^{E_{a}\left(\frac{1}{kT_{c}}-\frac{1}{kT}\right)}$$

where $E_{a}$ characterises the change in $gp_{i}\left(T_{c}\right)$ with temperature owing to temperature‐driven selection. Substituting the temperature dependence for $gp_{i}\left(T_{c}\right)$ into eqn (6) and simplifying, yields the following expression for the temperature dependence of GPP,

(8) $$GPP_{s}\left(T\right)=GPP\left(T_{c}\right)e^{E_{a}+E_{gp}\left(\frac{1}{kT_{c}}-\frac{1}{kT}\right)}$$

Under the ‘hotter‐is‐better’ model of thermal adaptation (Box 1 & Fig. 1a), where a single activation energy governs the temperature dependence of metabolism within and across species (Gillooly *et al*. 2001; Savage *et al*. 2004; Angilletta *et al*. 2010) and $E_{a}=0\;\mathrm{eV}$, the ecosystem‐level temperature dependence would equal that of individual‐level metabolism (i.e. $E_{GPP}=E_{gp}$; Box 1 & Fig. 1d (1)). This is the typical assumption made in metabolic theory (Allen *et al*. 2005; Demars *et al*. 2016). However, when $E_{a}\neq 0\;\mathrm{eV}$, $E_{GPP}=E_{a}+E_{gp}$, and the ecosystem‐level temperature‐dependence will deviate from the average organism‐level temperature‐dependence owing to the effects of temperature‐driven selection on $gp_{i}\left(T_{c}\right)$. If selection results in complete compensation (i.e. $E_{a}=-E_{gp}$; Fig. 1b), and $M_{s}$ does not covary with temperature, then ecosystem‐level GPP will be independent of temperature (i.e. $E_{GPP}=0\;\mathrm{eV}$; Fig. 1d (2)). Following the same reasoning, any temperature‐dependence in $M_{s}$ will also result in deviations from the average organism‐level activation energy. For example recent experimental work has shown that covariance between temperature and rates of nutrient cycling can cause $M_{s}$ to increase with temperature (Welter *et al*. 2015; Williamson *et al*. 2016), $M_{s}\approx e^{E_{b}\left(\frac{1}{kT_{c}}-\frac{1}{kT}\right)}$, where $E_{b}$ characterises the temperature dependence of total autotrophic biomass. When $E_{b}>0\;\mathrm{eV}$, substituting in the temperature‐dependence for $M_{s}$ into eqn (8) leads to an increase in the ecosystem‐level temperature‐dependence regardless of the effects of temperature‐driven selection ($E_{GPP}=E_{gp}+E_{b}+E_{a}$; Fig. 1d (3)). This model emphasises how different ecological and evolutionary mechanisms that drive temperature‐dependent variation in organism‐level metabolic traits and/or ecosystem biomass pools can influence the emergent long‐term temperature sensitivity of ecosystem metabolism (Box 1; Fig. 1c, d).

We now use measurements of the temperature‐dependence of organism‐ and ecosystem‐level photosynthesis from a catchment of naturally warmed geothermal streams to test the expectations of our model and investigate how ecological and evolutionary processes shape the long‐term temperature sensitivity of GPP. Critically, this system allows us to measure photosynthetic responses to temperature at both organism and ecosystem scales from sites that are in close proximity, yet differ substantially in their thermal history (i.e. 20 °C *in situ* temperature gradient among sites).

## Methods

The study was conducted in a geothermally active valley close to Hveragerði village, 45 km east of Reykjavík, Iceland (64.018350, −21.183433). The area contains a large number of mainly groundwater‐fed streams that are subjected to differential natural geothermal warming from the bedrock (O'Gorman *et al*. 2014). Twelve streams have been mapped in the valley with average temperatures ranging from 7 to 27 °C (Fig. S1 & Table S2). We measured a number of physical and chemical variables across the catchment (Table S4) and none of these variables were significantly correlated with temperature (Table S5). The study was carried out during May and June in 2015 and 2016.

To measure the organism‐level metabolic thermal response, we sampled 13 of the most abundant macroscopic cyanobacteria, filamentous eukaryotic algae, and bryophyte taxa from eight streams spanning the catchment's full thermal gradient. Because we sampled macroscopic algae – e.g. crops of filamentous algae or bryophyte fronds – measurements of metabolic rate are assumed to be at the level of the focal organism. We acknowledge that commensal microbes (e.g. protists and bacteria) are likely to be associated with these samples, but we assume that these organisms contribute a tiny fraction of the total biomass relative to the focal organism. Given the sensitivity of the O_2_ electrode used to characterise the metabolic thermal responses, these commensal organisms likely make a negligible contribution to the measurements of metabolism. For each focal organism, we first characterised a photosynthesis‐irradiance (PI) curve at the average temperature of the stream from which it was sampled. Net photosynthesis was measured as O_2_ evolution in the light and respiration as O_2_ consumption in the dark immediately after the light response. We estimated the optimal light intensity for net photosynthesis from the resulting PI curve using a modification of Eilers’ photoinhibition model (Eilers & Peeters 1988) (see supplementary methods and Fig. S2). The optimum light intensity ($I_{opt}$, μmol^−1^ m^−2^ s^−1^) for each taxon was then used for measuring net photosynthesis at all other assay temperatures in the acute thermal gradient experiments. Instantaneous rates of net photosynthesis (at $I_{opt}$) and respiration were then taken at temperatures ranging from 5 to 50 °C. Rates of gross photosynthesis were calculated by summing rates of net photosynthesis and respiration (see supplementary methods for a full description of the protocols for measuring the organism‐level thermal response).

Rates of photosynthesis and respiration were normalised to biomass by expressing each rate measurement per unit of chlorophyll *a*. Acute temperature responses of chlorophyll‐normalised gross and net photosynthesis and respiration were fitted to the modified Sharpe–Schoolfield equation for high‐temperature inactivation (eqn (1)). Best fits for each thermal response curve were determined using nonlinear least squares regression using the ‘nlsLM’ function in the ‘minpack.lm’ (Elzhov *et al*. 2009) package in R statistical software (R Core Team 2014; v3.2.2), following the methods outlined in Padfield *et al*. (2016).

We tested for temperature‐driven selection on metabolic traits by assessing whether the parameters in eqns (1) and (2), as well as the rate of gross photosynthesis at the average temperature of the natal stream of the focal organism, $gp\left(T_{s}\right)$ *,* varied systematically with temperature across the catchment. We fitted the metabolic traits to a modified Boltzmann–Arrhenius function within a linear mixed effects modelling framework:

(9) $$\ln z\left(T\right)=\ln z\left(T_{c}\right)+E_{a}\left(\frac{1}{kT_{c}}-\frac{1}{kT}\right)+\varepsilon^{t}$$

where *z* is the metabolic trait at stream temperature, T, $z(T_{c})$ is the value of the trait at the mean temperature across all streams, $T_{c}$, $E_{a}$ is the activation energy that determines how much *z* changes as a function of T due to temperature‐driven selection and $\varepsilon^{t}$ is a random effect on the intercept accounting for multiple measurements of the same metabolic trait of each focal organism (i.e. one value each for gross and net photosynthesis and respiration). We fitted eqn (9) to each metabolic trait with stream temperature, flux (three‐level factor with ‘gross’ and ‘net photosynthesis’ and ‘respiration’) and their interaction as fixed effects (Table S7). Significance of the parameters was determined using likelihood ratio tests. Model selection was carried out on models fitted using maximum likelihood and the most parsimonious model was refitted using restricted maximum likelihood for parameter estimation.

Ecosystem‐level metabolism was calculated from measurements of dissolved oxygen over time in each stream using the single‐station method (Odum 1956). Dissolved oxygen concentration and temperature were monitored at 1‐min intervals (Figs S3 and S5). Light sensors were deployed simultaneously at two sites in the centre of the catchment. Rates of gross primary productivity, GPP, were estimated using the single‐station method using a framework based on Odum's O_2_ change technique (Odum 1956). At the end of the 2 years of sampling, we had 39 daily estimates of GPP across the 15 sites (Table S3; see supplementary methods for a full description of the protocols for estimating GPP).

In 2016, we also measured autotrophic biomass density (g Chl *a *m^−2^) across the catchment by taking measurements of chlorophyll *a*. Because our aim was to determine the coupling between biomass and GPP, we used chlorophyll *a* as a proxy for the total photosynthetically active fraction of the biomass pool. The total autotrophic biomass, $M_{s}$, of each stream reach was estimated by multiplying average autotrophic biomass density by the total reach area, which was estimated from the mean width and the upstream distance the oxygen sensor integrated over (Chapra & Di Toro 1991; Demars *et al*. 2015; see supplementary methods). Biomass‐corrected rates of GPP per stream (g O_2_ g Chl *a* ^−1^ day^−1^) were calculated by dividing areal rates of GPP by the total autotrophic biomass, $M_{s}$, in the upstream reach.

We used linear mixed‐effects modelling to investigate the temperature‐dependence of GPP across the catchment, allowing us to control for the hierarchical structure of the data (e.g. variance of days nested within years nested within streams). We characterised the temperature‐dependence of GPP with a linearised version of the Boltzmann–Arrhenius function in a linear mixed effects model:

(10) $$\ln GPP_{s}\left(T\right)=E_{GPP}\left(\frac{1}{kT_{c}}-\frac{1}{kT}\right)+\left(\ln GPP\left(T_{c}\right)+\varepsilon_{P}^{s/y/d}\right)$$

where $GPP_{s}\left(T\right)$ is the rate of GPP in stream *s* on year *y* on day *d* at temperature *T* (Kelvin), $E_{GPP}$ is the activation energy (eV) which characterises the exponential temperature sensitivity of GPP, $\ln GPP\left(T_{c}\right)$ is the average rate of GPP across streams and days normalised to $T_{c}$ = 283 K (10 °C) and $\varepsilon_{P}^{s/y/d}$ is a nested random effect that characterises deviations from $\ln GPP\left(T_{c}\right)$ at the level of *d* within *y* within *s*. Significance of the parameters and model selection were carried out as described above for the analysis of the organism‐level metabolic traits (Table 1).

**Table 1:** Results of the linear mixed effects model analysis for gross primary productivity (GPP) for all years and 2016 only

Model	d.f.	AICc	log lik	L‐ratio	*P*
**All years:**					
Random effects structure					
Random = 1 | stream/year/day					
Fixed effects structure					
**1. ln GPP ~ 1 + stream temperature**	**6**	**82.9**	**−34.0**		
2. ln GPP ~ 1	5	85.8	−36.9	5.80	**0.016**
**2016 only:**					
Random effects structure					
Random = 1 | stream/day					
Fixed effects structure					
1. ln GPP ~ 1 + stream temperature + ln biomass	6	48.8	−14.9		
**2. ln GPP ~ 1 + ln biomass**	**5**	**45.3**	−**15.3**	0.87	0.35
3. ln GPP ~ 1	4	45.8	−17.4	4.25	**0.04**

Notes

The results of the model selection procedure on the fixed effect terms are given and the most parsimonious models are highlighted in bold. Analyses reveal that *in situ* GPP increased significantly with stream temperature. The analyses for 2016 show that the observed temperature response was driven by covariance between biomass and temperature rather than the direct effects of temperature on rates of photosynthesis *per se*.

We tested for the effect of total autotrophic biomass and temperature on *in situ* GPP across the catchment using the data from 2016 (where we also quantified autotroph biomass) by undertaking a multiple regression by expanding eqn (2) to include the effect the biomass:

(11) $$lnGPP_{s}\left(T\right)=E_{GPP}\left(\frac{1}{kT_{c}}-\frac{1}{kT}\right)+\beta \ln M_{s}+\left(\ln GPP\left(T_{c}\right)+\varepsilon_{P}^{s/d}\right)$$

where β characterises the power‐law scaling of $GPP_{s}\left(T\right)$ with $M_{s}$ and the random effects specification was changed to account for deviations from $\ln GPP\left(T_{c}\right)$ between days nested within streams. Model selection was as described above (Table 1). For additional information on the study site, sampling and estimation methods, see supplementary methods.

## Results

### Temperature‐driven selection on metabolic traits

**Figure 2:**
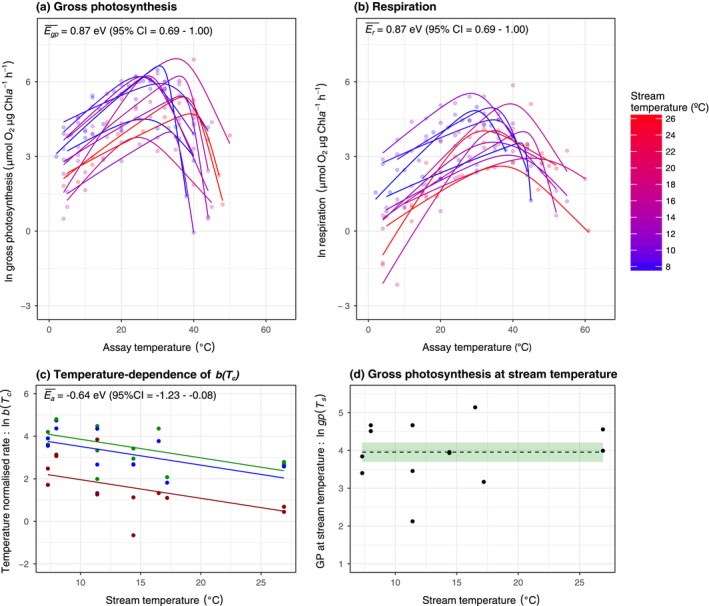
Temperature‐driven shifts in metabolic traits. (a,b) Acute thermal response curves for gross photosynthesis and respiration were measured for each isolated autotroph from streams spanning average temperatures from 7 °C (blue) to 27 °C (red). Fitted lines are based on the best‐fit parameters from non‐linear least squares regression using the modified Sharpe–Schoolfield model (see Methods). (c) Metabolic rates normalised to 10 °C, *b*(*T*_*c*_), decrease exponentially with increasing stream temperature for gross photosynthesis (green), net photosynthesis (blue) and respiration (red) (d) Rates of gross photosynthesis at the average stream temperature showed no temperature dependence. Fitted lines in (c) and (d) and coloured bands in (d) represent the best fit and the uncertainty of the fixed effects of the best linear mixed effect model.

Organism‐level gross photosynthesis and respiration followed unimodal responses to acute temperature variation and were well fit by eqn (1) (Fig. 2a,b). We predicted exponential declines in the metabolic normalisation constants, moving from cold to warm environments, owing to the effects of temperature‐driven selection. Consistent with this hypothesis, the log‐transformed rates of gross photosynthesis, $\left(\ln gp\left(T_{c}\right)\right)$ and respiration $\left(\ln r\left(T_{c}\right)\right)$ normalised to a reference temperature, $T_{c}$ = 10 °C, declined linearly with increasing stream temperature with the same temperature dependence (*E*_*a*_ = −0.64 eV; 95% CI: −1.22 to −0.05 eV; Fig. 2c). Since $np\left(T_{c}\right)=gp\left(T_{c}\right)-r\left(T_{c}\right)$, the normalisation for net photosynthesis also declined with increasing temperature with an *E*_*a*_ = −0.64 eV.

Because the dominant autotroph taxa varied across the streams (Table S6), the decline in the photosynthetic trait, $gp(T_{c})$, with increasing stream temperature is likely influenced by selection operating on trait variation both within and among taxa. To explore the effects of temperature‐driven selection within taxa, we analysed data from only the most common taxon, cyanobacteria from the genus *Nostoc* spp.*,* which were distributed across five streams spanning a gradient of 10.2 °C. $gp(T_{c})$, $np\left(T_{c}\right)$ and $r(T_{c})$ also decreased with increasing stream temperature in *Nostoc* spp. with the thermal sensitivity not significantly different from that of all the autotroph taxa together (Fig. S6). An important consequence of the decrease in $gp(T_{c})$ with increasing stream temperature was that rates of gross photosynthesis at the average temperature of each stream, $gp(T_{s})$, were independent of temperature across the catchment's thermal gradient (Fig. 2d), suggesting that temperature‐driven selection on photosynthetic traits led to complete temperature compensation of organism‐level metabolism.

Both the optimum temperature, $T_{opt}$, and $T_{h}$, which is the temperature at which half the enzymes are inactivated, were positively correlated with average stream temperature (Table S7) providing further evidence that each taxon was locally adapted to its natal thermal regime. We found no evidence for systematic variation in the activation or inactivation energies (E or $E_{h}$) across the thermal gradient, suggesting these traits are unlikely to be under strong selection (Table S7). Previous work has shown that photosynthesis has a lower activation energy than respiration (Allen *et al*. 2005; López‐Urrutia *et al*. 2006; Padfield *et al*. 2016). In contrast, we found that the average temperature sensitivities of gross photosynthesis and respiration were not significantly different and could be characterised by a common activation energy (*E* = 0.87 eV; 95% CI = 0.77 to 0.97 eV). Similarly, $E_{h}$, which characterises inactivation of kinetics past the optimum, was not significantly different between fluxes and could be characterised by a common value for respiration and photosynthesis (*E*_*h*_ = 4.91 eV; 95% CI: 3.95–5.97 eV).

### Ecosystem‐level gross primary productivity

**Figure 3:**
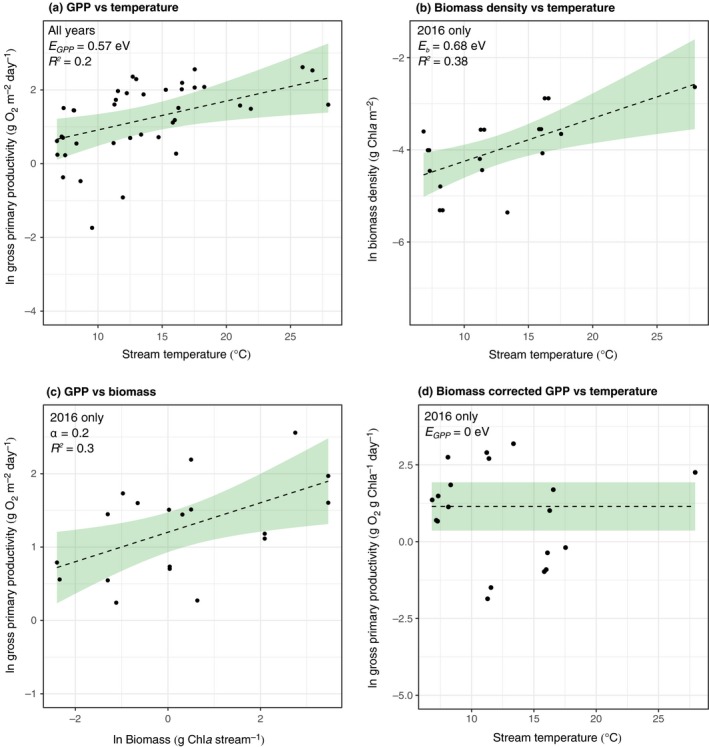
The effects of temperature and autotrophic biomass on gross primary productivity. Gross primary productivity (a) and autotrophic biomass density (b) increase with temperature across the catchment. (c) A multiple regression shows that variation in *in situ* GPP is driven primarily by changes in autotroph biomass. (d) After accounting for biomass, rates of biomass‐corrected GPP are invariant with respect to temperature across the catchment. Fitted lines in (a, c, d) represent the best fit and the uncertainty of the fixed effects of the best linear mixed effect model (Table 1). In (b) the lines represent the fitted line and associated confidence interval of a linear regression.

Based on the observation that the activation energies of gross photosynthesis ($E_{gp}$) and the parameter describing the temperature‐driven changes in $gp(T_{c})$, ($E_{a}$), were similar, but of opposite sign, the model for the scaling of metabolism from organisms to ecosystems (eqn (8)) predicts that rates of *in situ* GPP should be independent of temperature across the catchment (e.g. $E_{GPP}=E_{gp}+E_{a}\approx 0\;\mathrm{eV}$), provided that biomass does not covary with temperature. Rates of GPP increased with temperature and the long‐term temperature sensitivity of GPP yielded an activation energy of $E_{GPP}$ = 0.57 eV (95% CI: 0.10–1.04 eV; Fig. 3a).

To investigate potential covariance between temperature and biomass and its impact on the temperature‐dependence of GPP, in 2016 we also quantified total autotrophic biomass. Autotroph biomass density increased systematically with temperature across the catchment with a temperature sensitivity of $E_{b}$ = 0.68 eV (95% CI: 0.24–1.12 eV; Fig. 3b). The similarity between $E_{GPP}$ and $E_{b}$ – they have 95% confidence intervals that overlap – indicates that covariance between autotrophic biomass and temperature could be the main driver of the temperature dependence of GPP across the catchment.

We quantified the effects of both temperature and autotroph biomass, $M_{s}$, on GPP using multiple regression in a mixed effects modelling framework for data collected in 2016 (see Methods). The best fitting model included only $\ln(M_{s})$ as a predictor (Table 1; Fig. 3c) and after controlling for variation in $\ln(M_{s})$, rates of biomass‐specific GPP were independent of temperature across the catchment (Table 1; Fig. 3d). These findings are consistent with predictions from our model and provide evidence that systematic variation in the photosynthetic normalisation constant owing to temperature‐driven selection results in complete compensation of biomass‐specific metabolic rates at organism and ecosystem scales.

## Discussion

Understanding how ecosystem‐level properties like GPP will respond to global warming is of central importance to predicting the response of the carbon cycle and contributing biogeochemical and food web processes to climate change. It is, however, a major challenge that requires an integration of physiological, ecological and evolutionary processes that together shape the emergent response of ecosystem metabolism to long‐term changes in temperature. We have addressed this key problem by extending the general model for ecosystem metabolism from metabolic scaling theory (Enquist *et al*. 2003, 2007; Allen *et al*. 2005; Kerkhoff *et al*. 2005) and testing its predictions at organism and ecosystem scales in a catchment of naturally warmed geothermal streams. Our model and analyses demonstrate that temperature‐driven selection on metabolic traits and shifts in ecosystem biomass can be as important as the direct effects of temperature on metabolism in shaping the temperature dependence of GPP.

Our model predicted that when the temperature‐dependence of the metabolic normalisation constant across taxa inhabiting environments with different thermal histories is inversely proportional to that of organism‐level metabolism, the two temperature sensitivities cancel, rendering biomass‐specific metabolic rates independent of temperature. Measurements of the thermal response curves for photosynthesis and respiration from the autotrophs isolated across the 20 °C *in situ* gradient provided strong support for this prediction, with rates of gross photosynthesis independent of temperature across the catchment's thermal gradient. In addition, activation energies characterising the temperature‐dependence of organism‐level gross photosynthesis and the photosynthetic normalisation, $gp(T_{c})$, were similar in magnitude but of opposite sign.

The exponential decline in $gp\left(T_{c}\right)$ along the *in situ* thermal gradient primarily reflected turnover in the composition of the dominant autotroph taxa across the streams resulting from temperature‐driven selection on trait variation among taxa (e.g. species sorting). This result is in line with work demonstrating declines in the metabolic normalisation constant across vascular plant species along broad‐scale latitudinal gradients in terrestrial ecosystems (Atkin *et al*. 2015). However, we also found a comparable negative temperature dependence of $gp(T_{c})$ within the most common and widely distributed genus, *Nostoc* spp., indicating that temperature‐driven selection within taxa was also an important determinant of variation in this key trait among sites in our study. This finding is consistent with work demonstrating down‐regulation of the metabolic normalisation constant in a unicellular alga via rapid (e.g. over 100 generations or 45 days) evolutionary adaptation to an experimental thermal gradient in the laboratory (Padfield *et al*. 2016). Collectively, this work highlights that changes in the metabolic normalisation constant result from temperature‐driven selection both within and across taxa and can give rise to complete temperature compensation of metabolic capacity over broad thermal gradients (Fig. 1b).

Our work shows that temperature‐driven selection, in driving complete temperature compensation of organism‐level metabolism, had important implications for understanding the temperature dependence of ecosystem‐level GPP across the catchment. GPP increased with temperature across the catchment (Fig. 3a) with a temperature dependence equal to another recent study on metabolism in geothermal streams (Demars *et al*. 2016), but it did so because biomass also positively covaried with temperature (Fig. 3b). This is likely driven by a shift in algal community composition, with warmer streams being dominated by cyanobacteria capable of fixing nitrogen, alleviating the constraints imposed by the limiting concentrations of inorganic nitrogen observed in these streams (Table S4) (Welter *et al*. 2015; Williamson *et al*. 2016). After accounting for covariance with biomass, biomass‐specific GPP was independent of temperature (Fig. 3c), consistent with the effects of temperature compensation of organism‐level metabolism. These findings confirm the predictions of our model and previous suggestions (Kerkhoff *et al*. 2005; Enquist *et al*. 2007) that local adaptation and species sorting can yield the paradoxical phenomenon that rates of biomass‐specific ecosystem metabolism are independent of temperature over thermal gradients that have been maintained over long timescales.

A great deal of empirical and theoretical work is still required to develop a complete, general theory that predicts how ecosystem properties emerge from ecological and evolutionary processes. Our work adds to recent efforts to this end (Enquist *et al*. 2007; Yvon‐Durocher & Allen 2012; Smith & Dukes 2013; Daines *et al*. 2014; Schramski *et al*. 2015; Smith *et al*. 2016) by showing how the temperature‐dependence of ecosystem biomass and the organism‐level photosynthetic normalisation constant alter the emergent temperature sensitivity of ecosystem‐level GPP. One important gap in the theory presented here is a mechanistic model for the temperature dependence of the metabolic normalisation constant owing to temperature‐driven selection. Our representation in eqn (7) is merely a statistical description of an empirical phenomenon. The metabolic cold‐adaptation hypothesis seeks to explain the observation that species from cold environments often have higher mass‐specific metabolic rates compared to counterparts from warmer regions as an evolutionary adaptation to compensate for lower biochemical reaction rates (Addo‐Bediako *et al*. 2002). However, a quantitative, first‐principles derivation of this pattern remains elusive. Recent work on autotrophs has proposed that down‐regulation of respiration rates as organisms adapt to warmer environments is driven by selection to maintain the carbon‐use efficiency above a threshold when rates of respiration are more sensitive to temperature than those of photosynthesis (Padfield *et al*. 2016). Yet, as we have shown here, the assumption that the activation energy of respiration is always larger than that of photosynthesis does not always hold.

A better understanding of the mechanisms that give rise to the emergence of ecosystem properties is central to improving predictions of how global warming will alter the feedbacks between the biosphere and the carbon cycle (Levin 1998; Ziehn *et al*. 2011; Booth *et al*. 2012). Incorporating ecological changes in community biomass and evolutionary shifts in metabolic traits into earth system and ecosystem models should be considered as a priority (Smith & Dukes 2013; Daines *et al*. 2014; Smith *et al*. 2016), especially in light of our finding that these indirect effects of temperature can be of similar magnitude to the direct effects of temperature on physiological rates.

We capitalised on a ‘natural experiment’ using a geothermally heated stream catchment to show that temperature‐driven selection on photosynthetic traits results in an equivalence in biomass‐normalised GPP over a 20 °C *in situ* temperature gradient. Our results suggest that temperature‐driven selection on metabolic traits within and among taxa plays a key role in determining how metabolic rates scale from populations to ecosystems, questioning the assumption that the effects of temperature on enzyme kinetics can be applied directly to assess the long‐term effects of temperature on ecosystem metabolism (Demars *et al*. 2016). They also shed light on the way in which the interplay between ecological and evolutionary processes could influence the response of the carbon cycle, and hence constituent food web and biogeochemical processes, to future environmental change.

## Author Contributions

GY‐D and CL conceived the study. DP and GY‐D designed the experimental work and DP, GY‐D, CL, ES and the student research team conducted the experiments. DP and GY‐D analysed the data and all authors contributed to writing the paper. The authors declare no conflict of interest.

## Data Availability

All data and code to reproduce the analyses in the paper can be accessed at https://git.io/v5TUG
