# Supplementary material for: Metabolic compensation constrains the temperature dependence of gross primary production
Source: Ecol Lett. 2017 Aug 29;20(10):1250–60. doi: 10.1111/ele.12820 (PMC6849571; doi:10.1111/ele.12820)
Supplement: Supplementary file 1 [file ELE-20-1250-s001.pdf]

SUPPLEMENTARY INFORMATION

for

Metabolic compensation constrains the temperature dependence of gross primary production

Daniel Padfield[1], Chris Lowe[1,2*], Angus Buckling[1,2], Richard Ffrench-Constant[2], Elisa Schaum[1], Student Research Team[2], Simon Jennings[3,4,5], Felicity Shelley[6], Jón S. Ólafsson[7] & Gabriel Yvon-Durocher[1*]

Author affiliations:

[1] Environment and Sustainability Institute, University of Exeter, Penryn, Cornwall, TR10 9EZ, U.K.

[2] Centre for Ecology and Conservation, College of Life and Environmental Sciences, University of Exeter, Penryn, Cornwall, TR10 9FE, U.K.

[3] Centre for Environment, Fisheries and Aquaculture Science, Lowestoft, NR33 0HT, U.K.

[4] School of Environmental Sciences, Norwich Research Park, University of East Anglia, Norwich, NR4 7TJ, U.K.

[5] International Council for the Exploration of the Sea, H. C. Andersens Boulevard 44-46, 1553 Copenhagen V, Denmark.

[6] School of Biological and Chemical Sciences, Queen Mary University of London, London, E1 4NS, U.K.

[7] Marine and Freshwater Research Institute, Árleyni 22, 112 Reykjavik, Iceland.

Corresponding authors: Gabriel Yvon-Durocher (g.yvon-durocher@exeter.ac.uk) or Chris Lowe (c.lowe@exeter.ac.uk)

Section 1. Supplementary Tables and Figures

Table S1: Parameters used in the formulation of metabolic scaling theory.

Notation	Formulation	Description	Units
**Organism-level**			
E_{gp}		Activation energy of gross photosynthesis	eV
E_{np}		Activation energy of net photosynthesis	eV
E_r		Activation energy of respiration	eV
E_h		Inactivation energy	eV
$gp(T_c)$		Temperature normalised rate of gross photosynthesis	µmol O_2 µg $Chla^{-1}$ h^{-1}
$np(T_c)$		Temperature normalised rate of net photosynthesis	µmol O_2 µg $Chla^{-1}$ h^{-1}
$r(T_c)$		Temperature normalised rate of respiration	µmol O_2 µg $Chla^{-1}$ h^{-1}
T_{opt}		Optimum temperature	ºC
T_h		Temperature where half of enzymes are inactivated	ºC
m^a		Mass dependence of metabolic rate	µg $Chla^{-1}$
**Ecosystem-level**			
$GPP(T_c)$	$\frac{1}{A}\sum_{i=1}^{J} gp_i(T_c)\, m_i^{\alpha}$	Temperature normalised rate of gross primary productivity	g O_2 g $Chla^{-1}$ day^{-1}
E_{GPP}	$E_{gp} + E_a + E_b$	Activation energy of gross primary productivity	eV
M_s	$\frac{1}{A}\sum_{i=1}^{J} m_i$	Total biomass	g Chla m^{-2}
E_a		Activation energy of adaptation of $gp(T_c)$	eV
E_b		Activation energy for temperature dependence of biomass	eV

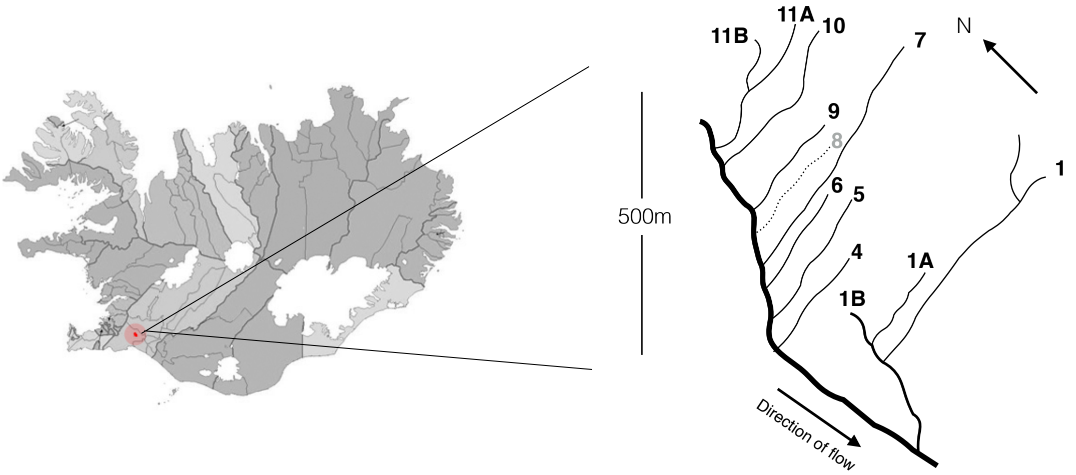

Figure S1. Map of the geothermal stream system in a valley near Hveragerdi, SW Iceland (64.018350, -21.183433).

Table S2. Mean, minimum and maximum temperature values averaged across days and years (May 2015, May 2016) in the 15 sites. Values are based on a temperature estimates taken at 1 minute intervals. The sites are listed with increasing mean temperature.

	Temperature (ºC)		
Site	Mean	Minimum	Maximum
S9	6.8	5.3	7.9
S7 : high	7.1	6.7	7.9
S4	7.3	5.1	8.9
S1A	8	4.5	11.8
S1B	8.2	7.1	9.7
S6	11	7.3	14.1
S7 : low	11.4	10.4	12.1
S1 : low	12.1	9.7	16.3
S5 : low	13.2	12.1	14.8
S10	14.4	10.4	16.9
S11A	14.4	12.4	16.6
S1 : high	16.5	13.3	18.8
S11B : high	17.2	14.7	19.6
S11B : low	21.5	19.8	23.4
S5 : high	26.9	24.8	28.6

Table S3. The number of days of stream gross primary productivity measured from each site across years. Sites are ordered by increasing average stream temperature

	Number of days of site GPP measurements		
Site	2015	2016	Total
S9	0	1	1
S7 : high	2	2	4
S4	0	1	1
S1A	2	1	3
S1B	0	2	2
S6	2	1	3
S7 : low	0	1	1
S1 : low	2	2	4
S5 : low	0	1	1
S10	2	1	3
S11A	2	2	4
S1 : high	2	1	3
S11B : high	2	2	4
S11B : low	2	0	2
S5 : high	2	1	3

Table S4. Key physical and chemical features of the 15 sites investigated

Site	width (m)	depth (m)	velocity (m s^{-1})	integrated upstream estimate (m)	pH	conductivity (µS m^{-1})	nutrients (µmol L^{-1})			
							NO_2	NO_3	NH_4	PO_4
S9	0.41	0.027	0.11	29	7.57	173.3	0.29	0.23	0.27	0.86
S7 : high	0.4	0.053	0.3	237	7.43	359.1	0.22	0.44	0.28	0.7
S4	0.46	0.06	0.36	191	7.27	204.6	0.2	0.08	0.22	0.14
S1A	0.59	0.07	0.5	561	7.40	230.9	0.25	0.4	0.7	0.54
S1B	0.42	0.058	0.14	131	7.50	462.4	0.28	0.25	0.18	0.17
S6	0.19	0.029	0.12	302	7.43	289.6	0.22	0.4	0.21	1.02
S7 : low	0.3	0.043	0.4	107	7.43	304.7	0.22	0.44	0.28	0.7
S1 : low	1.1	0.13	0.81	917	7.36	305.2	0.26	0.26	0.48	0.35
S5 : low	0.32	0.041	0.09	60	7.63	273.6	0.22	0.57	0.17	0.14
S10	0.22	0.109	0.24	552	7.53	167.0	0.35	-	0.24	0.74
S11A	0.71	0.078	0.77	405	7.17	235.7	0.24	0.29	0.19	0.55
S1 : high	0.74	0.12	0.61	884	7.20	321.7	0.26	0.26	0.48	0.35
S11B : high	0.31	0.042	0.33	222	7.33	407.9	0.25	0.25	0.27	1.25
S11B : low	0.4	0.042	0.33	370	7.33	407.9	0.25	0.25	0.27	1.25
S5 : high	0.17	0.037	0.06	109	7.63	319.2	0.22	0.57	0.17	0.27

Table S5. Pearson correlation coefficients between temperature and physical and chemical variables

Variable	*r*	P value
width	-0.14	0.56
depth	0.07	0.77
velocity	0.04	0.87
pH	-0.03	0.91
conductivity	-0.02	0.92
NO_2	-0.001	0.47
NO_3	0.18	0.47
NH_4	-0.19	0.44
PO_4	0.07	0.77

Table S6. The metabolic traits governing the thermal response curves for the autotroph taxa from each site.

			Net photosynthesis					Respiration					Gross photosynthesis				
Site	Year	Taxon	ln $np(T_c)$ (µmol O_2 µg Chla^{-1} h^{-1} @ 10ºC)	E_{np} (eV)	E_h (eV)	T_h (ºC)	T_{opt} (ºC)	ln $r(T_c)$ (µmol O_2 µg Chla^{-1} h^{-1} @ 10ºC)	E_r (eV)	E_h (eV)	T_h (ºC)	T_{opt} (ºC)	ln $gp(T_c)$ (µmol O_2 µg Chla^{-1} h^{-1} @ 10ºC)	E_{gp} (eV)	E_h (eV)	T_h (ºC)	T_{opt} (ºC)
S4	2016	Cladophora	3.9	1.03	4.39	30.6	28.48	2.48	1.01	3.78	31.66	29.53	4.2	0.92	9.19	32.49	30.57
S1A	2016	Cladophora	4.74	0.79	2.58	28.33	25.88	3.07	0.64	4.36	37.28	33.97	4.77	0.89	2.71	26.98	24.95
S1A	2016	Nostoc	4.37	0.52	8.77	36.87	34.28	3.14	0.44	4.26	38.83	34.63	4.8	0.47	2.72	35.36	30.71
S4	2015	Cladophora	3.55	0.87	1.78	21.67	21.52	1.71	0.45	17.21	43.78	41.97	3.61	0.53	2.18	30.18	26.1
S7 : high	2016	Cladophora	4.35	0.73	7.04	31.77	29.33	3.85	0.8	2.94	31.06	28.42	4.48	0.93	3.51	28.24	25.99
S7 : high	2016	Nostoc	2.67	0.98	3.57	34.32	32.1	1.27	0.93	1.77	34.12	34.59	3.33	0.62	9.05	38.83	36.43
S7 : high	2015	Feathermoss	1.32	0.77	4.99	34.19	31.44	1.26	0.55	2.13	42.8	38.64	1.99	0.66	8.81	35.6	33.28
S11A	2016	Nostoc	2.67	1.91	5.29	28.47	27.62	1.13	0.71	5.85	45.74	42.79	2.95	1.57	4.37	28.46	27.43
S10	2016	Nostoc	2.68	1.08	9.9	38.53	36.76	-0.66	1.64	3.15	34.74	34.94	3.42	0.85	7.12	38.39	36.06
S1 : high	2016	Nostoc	3.77	1.03	8.87	39.61	37.69	1.33	1.09	3.23	41.01	39.26	4.36	0.85	3.66	37.86	35.15
S11b : high	2015	Feathermoss	1.82	1.12	2.64	24.53	23.64	1.1	0.48	1.64	49.7	45	2.08	1.14	2.5	25.19	24.64
S5 : high	2015	Anabaena	2.58	0.54	5.9	42.5	39.19	0.68	0.66	2.04	39.71	36.65	2.73	0.55	5.69	42.37	39.02
S5 : high	2016	Anabaena	2.66	0.85	4.02	37.4	34.71	0.45	1.58	2.35	29.63	32.12	2.79	0.77	5.63	39.89	37.14

Table S7. Results of a linear effects model analysis for each metabolic trait with fixed effects of stream temperature and metabolic flux (see Methods). Significant models are highlighted in bold.

Metabolic Trait	Effect	d.f.	AIC	Log Lik	L-ratio	P value
$b(T_c)$	~ 1 + stream temperature * metabolic flux	8	96.94	-40.47		
	**~ 1 + stream temperature + metabolic flux**	6	94.89	-41.43	1.93	0.37
	~ 1 + metabolic flux	5	98.28	-44.14	5.41	**0.02**
E_a	~ 1 + stream temperature * metabolic flux	8	37.03	-10.51		
	~ 1 + stream temperature + metabolic flux	6	36.36	-12.18	3.33	0.189
	~ 1 + stream temperature	4	34.41	-13.21	2.05	0.36
	**~ 1**	3	32.92	-13.46	0.51	0.48
E_h	~ 1 + stream temperature * metabolic flux	8	72.92	-28.46		
	~ 1 + stream temperature + metabolic flux	6	73.83	-30.91	4.91	0.09
	~ 1 + metabolic flux	5	72.07	-31.04	0.24	0.62
	**~ 1**	3	71.37	-32.68	3.30	0.19
T_h	~ 1 + stream temperature * metabolic flux	8	-192.08	104.04		
	**~ 1 + stream temperature + metabolic flux**	6	-192.92	102.46	3.15	0.206
	~ 1 + metabolic flux	5	-190.32	100.16	4.60	**0.032**
T_{opt}	~ 1 + stream temperature * metabolic flux	8	-27.21	21.61		
	**~ 1 + stream temperature + metabolic flux**	6	-28.72	20.36	2.49	0.29
	~ 1 + metabolic flux	5	-24.54	17.27	6.18	**0.013**
$b(T_s)$	~ 1 + stream temperature * metabolic flux	8	48.64	-16.32		
	~ 1 + stream temperature + metabolic flux	6	44.68	-16.34	0.05	0.98
	**~ 1 + metabolic flux**	5	42.99	-16.49	0.31	0.58
	~ 1	4	64.21	-29.10	25.22	**<0.0001**

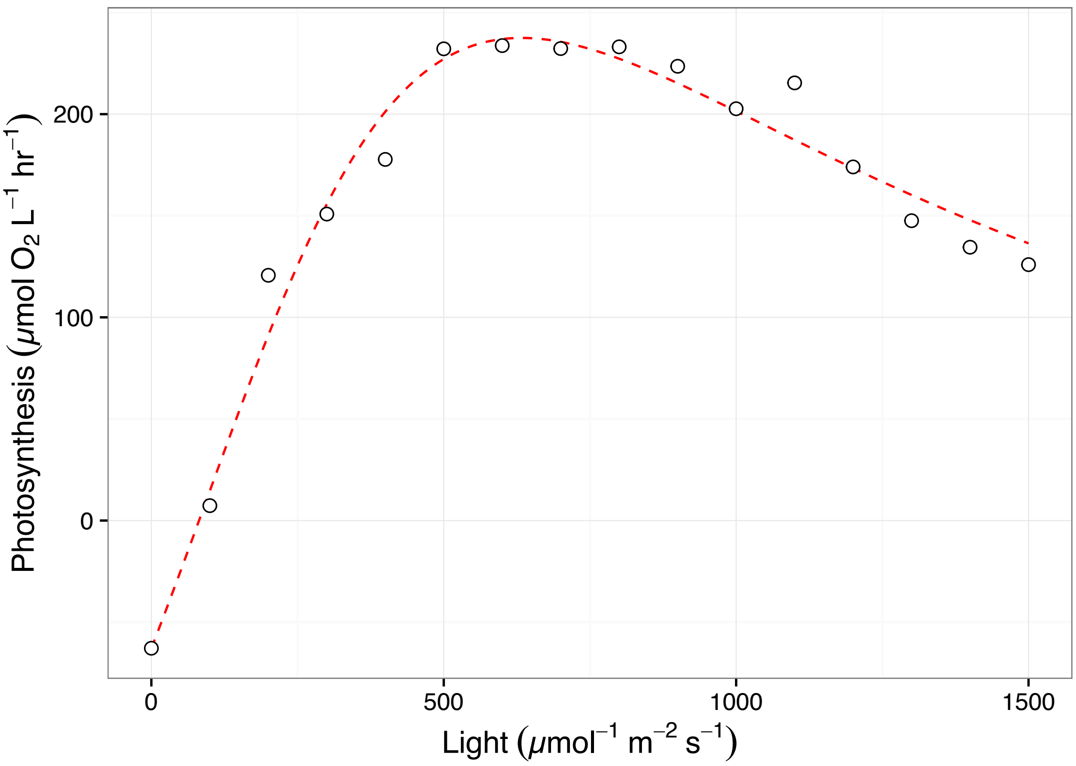

Figure S2. Photosynthesis irradiance curve used to determine optimal light for the acute temperature response of gross photosynthesis. Rates of net photosynthesis were measured at various light intensities at the average stream temperature of each biofilm. Here data are presented for *Nostoc spp.* in stream 7 (high) at 7.1 ºC. Lines represent the best fit to the modified Eiler's model using non-linear least squares regression (see Supplementary Methods).

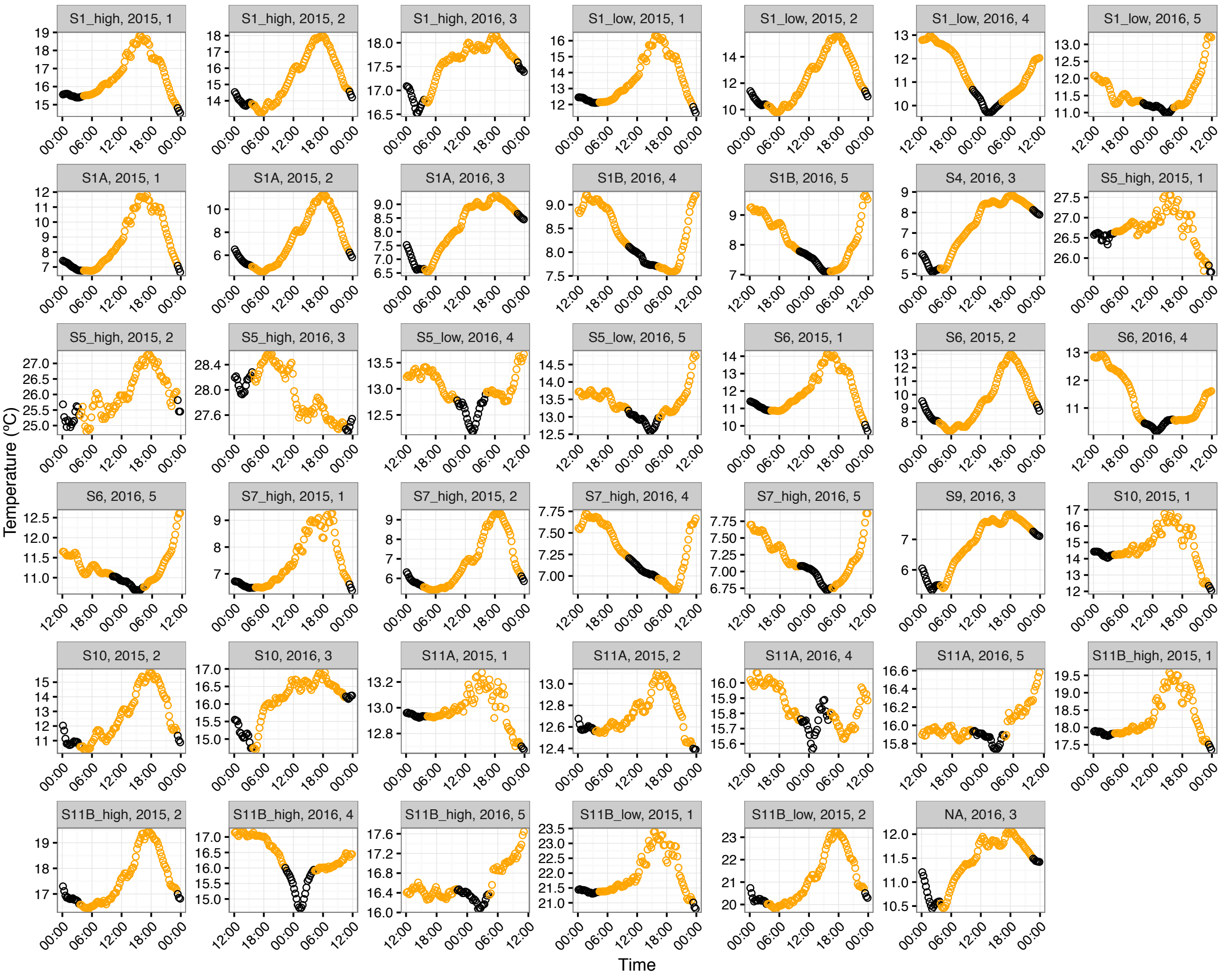

Figure S3. Daily cycles in temperature from each stream across days and years. Each panel is a single day of temperature variation split by each unique stream and across years (2015 or 2016). The data is split into "night" (black points) and "day" (yellow points) by defining night as < 5µmol m^{-2} s^{-1} (see Supplementary Methods).

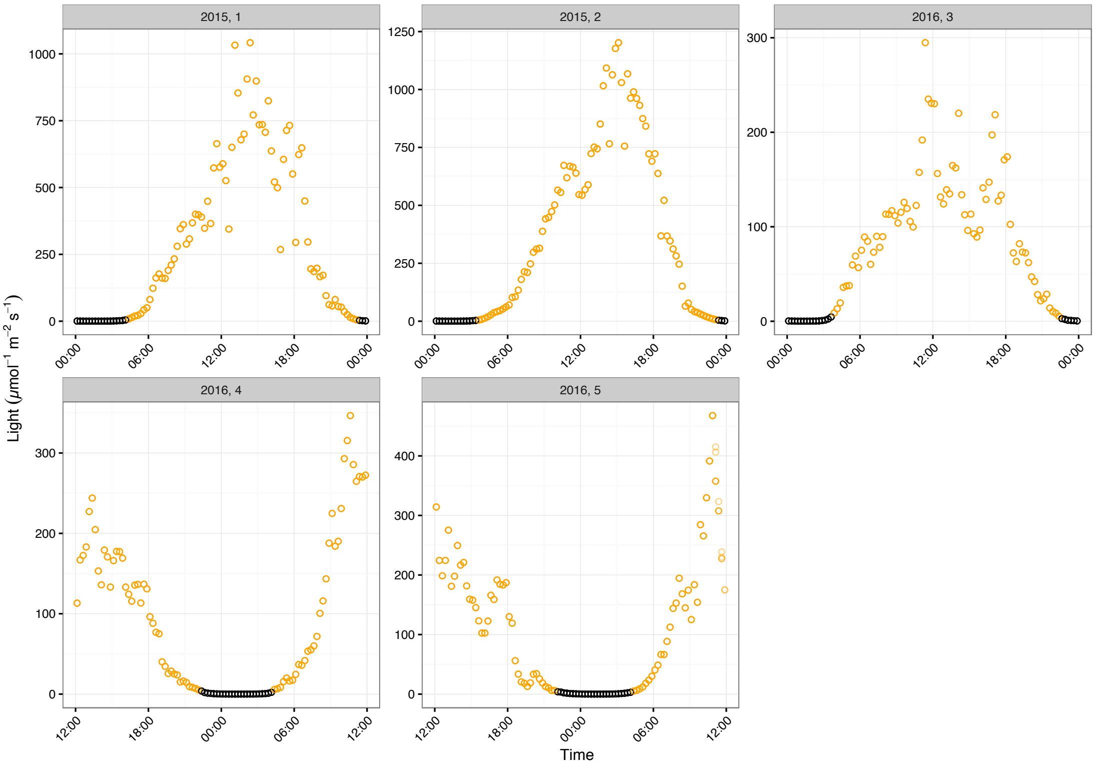

Figure S4. Daily cycles in light from across days and years. Each panel is a single day of light variation split by each unique stream and across years (2015 or 2016). The data is split into "night" (black points) and "day" (yellow points) by defining night as < 5µmol m^{-2} s^{-1} (see Supplementary Methods).

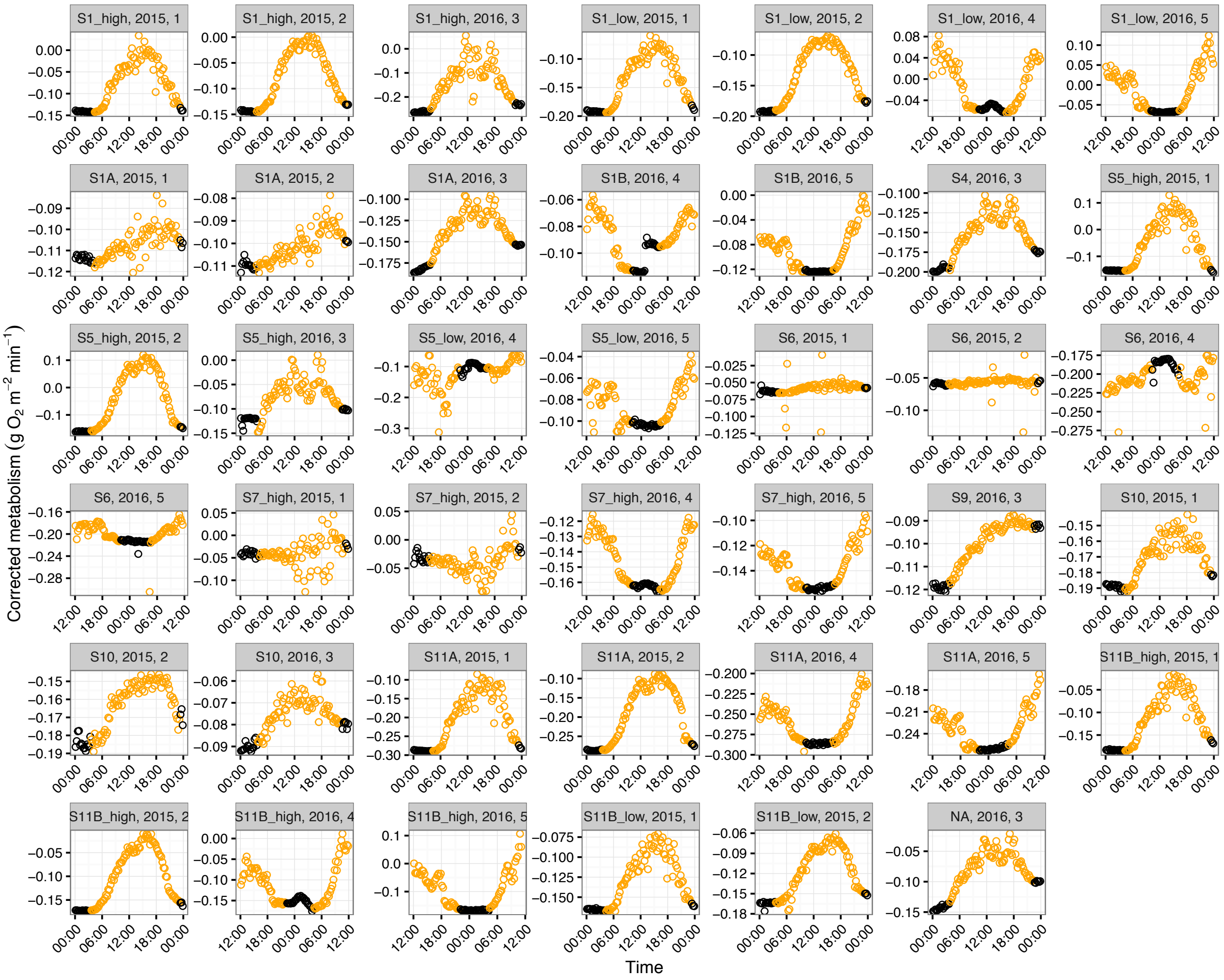

Figure S5. Daily cycles in metabolic flux from each site across days and years. Each panel is a single day of metabolic rate after accounting for reaeration (*ΔDO – G*; see Methods) split by each unique stream and across years (2015 or 2016). The data is split into "night" (black points) and "day" (yellow points) by defining night as < 5µmol m^{-2} s^{-1} (see Supplementary Methods).

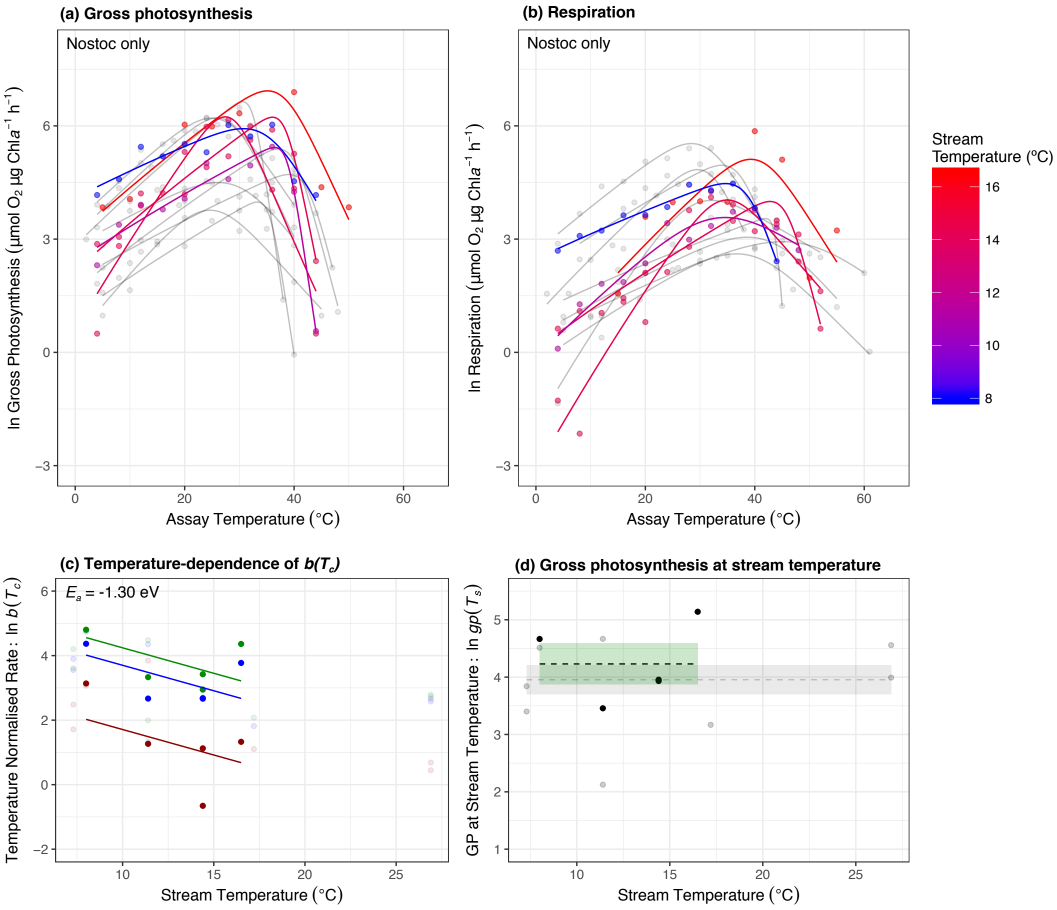

Fig S6. Patterns of thermal adaptation in *Nostoc* spp. only. (a,b) Acute thermal response curves for gross photosynthesis and respiration were measured for each isolated autotroph from streams spanning average temperatures from 7 ºC (blue) to 17 ºC (red) for stream biofilms dominated by *Nostoc* spp. (c) Metabolic rates normalised to 10 ºC, $b(T_c)$, decrease exponentially with increasing stream temperature for gross photosynthesis (green), net photosynthesis (blue) and respiration (red). (d) Rates of gross photosynthesis at the average stream temperature showed no temperature dependence. Grey points and lines highlight the other taxa to facilitate direct comparison to the relationship for *Nostoc* spp.

Section 2. Supplementary Methods.

Derivation of the activation energy of net photosynthesis. The rate of net photosynthesis, $np(T)$, at temperature, T, is equal to the difference between the rates of gross photosynthesis, $gp(T)$, and respiration, $r(T)$. Equation 5 (main text) implies that the temperature sensitivity of net photosynthesis will not follow a simple Boltzmann-Arrhenius relationship. Instead, the apparent activation energy of net photosynthesis, E_{np}, can be approximated in the vicinity of T_c as (Yvon-Durocher *et al.* 2014),

$$E_{np} \equiv \left.\frac{dln(np(T))}{d\left(\frac{1}{kT}\right)}\right|_{T=T_c} = \frac{E_{gp}\, gp(T_c) + E_r r(T_c)}{gp(T_c) + r(T_c)} \quad \text{(S1)}$$

which is equal to an average of the activation energies of E_{gp} and E_r, weighted by their respective normalisations, $gp(T_c)$ and $r(T_c)$. Using this approximation, we can then express the temperature dependence of np as

$$np(T) = np(T_c) m^{\alpha} e^{E_{np}\left(\frac{1}{kT_c} - \frac{1}{kT}\right)} \quad \text{(S2)}$$

where $np(T_c) = gp(T_c) - r(T_c)$. We quantified the accuracy of this approximation by comparing E_{np} derived using eq. S1 to the apparent activation energy of net photosynthesis measured by fitting eq. (1) to the net photosynthesis data (see Methods). The derived and measured estimates of E_{np}were positively correlated with a slope that had confidence intervals which overlapped unity (slope = 1.22, 95% CI: 0.78 – 1.65) and R^2 = 0.75 (Fig. S7).

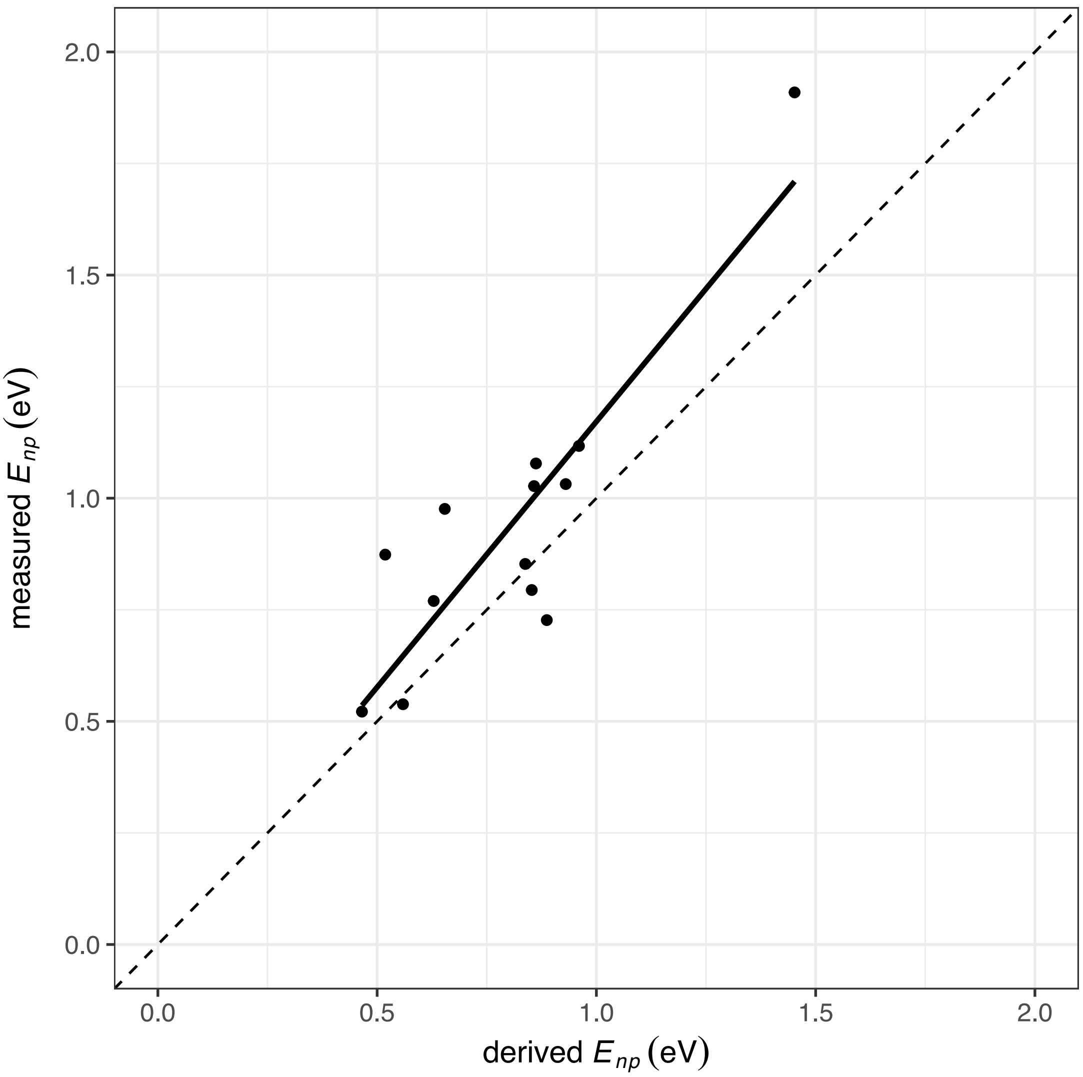

Figure S7. Comparison between measured and derived activation energies for net photosynthesis. Activation energies of net photosynthesis measured from fitting the rate data to the modified Sharpe-Schoolfield equation (eq. 1) correlate well with the derived activation energy of net photosynthesis calculated using equation S1. The fitted line is the best fit of a linear model and the 1:1 line is shown for comparison.

Additional methods on study site, measurements and estimation of organism- and ecosystem-level metabolism

Study site

The study was conducted in a geothermally active valley close to Hveragerdi village, 45 km east of Reykjavik, Iceland (64.018350, -21.183433). The area contains a large number of mainly groundwater-fed streams that are subjected to differential natural geothermal warming from the bedrock (O'Gorman *et al.* 2014). Twelve streams have been mapped in the valley with average temperatures ranging from 7 – 27 ºC (Fig. S1 & Table S2). We measured a number of physical (width, depth, velocity) and chemical (pH, conductivity, dissolved inorganic nutrients) variables across the catchment (Table S4) and none of these variables were significantly correlated with temperature (Table S5). The study was carried out during May and June in 2015 and 2016.

Inorganic nutrients

Water samples for measuring dissolved inorganic nutrient concentrations (NO_2^-, NO_3^-, NH_4^+ and PO_4^{3+}; µmol L^{-1}) were collected from each stream in 2016. Samples were filtered (Whatmann GF/F) and stored frozen at -20 ºC for subsequent analysis using a segmented flow auto-analyser (Table S4) (Kirkwood 1996).

Measuring the organism-level metabolic thermal response

We sampled 13 of the most abundant macroscopic cyanobacteria, filamentous eukaryotic algae, and bryophyte taxa from 8 streams spanning the catchment's full thermal gradient to characterise their metabolic thermal responses using an O_2

electrode system. Multiple taxa were sampled from four streams where more than one taxon was at high density (Table S6). Because we sampled macroscopic algae – e.g. crops of filamentous algae or bryophyte fronds – measurements of metabolic rate are assumed to be at the level of the focal organism. We acknowledge that commensal microbes (e.g. protists and bacteria) are likely to be associated with these samples, but we assume that these organisms contribute a tiny fraction of the total biomass relative to the focal organism. Given the sensitivity of the O_2 electrode, these commensal organisms likely make a negligible contribution to the measurements of metabolism. Rocks dominated by a focal alga were brought back to the laboratory and maintained in water from their natal stream over the course of the metabolic measurements. Metabolic rates were measured via changes O_2 concentration in a Clark-type oxygen electrode (Hansatech Ltd, King's Lynn UK Chlorolab2). For each incubation, a fresh sample of the focal organism was suspended in stream water from the natal stream filtered at 0.7 µm and placed in a gas tight cuvette (2 mL) associated with the O_2 electrode. The cuvette was surrounded by a water-jacket, connected to a recirculating water bath, which maintained a constant temperature. A magnetic stirrer within the cuvette ensured homogeneity of O_2 concentration throughout the chamber. For each focal organism, measurements first entailed characterising a photosynthesis-irradiance (PI) curve from 0 – 2000 µmol m^{-2} s^{-1} at the average temperature of the stream from which it was sampled. Net photosynthesis (np) was measured as O_2 evolution. Light intensities were maintained for one minute and were increased in intervals of 50 µmol^{-1} m^{-2} s^{-1} up to 300 µmol^{-1} m^{-2} s^{-1}, and then in intervals of 100 µmol^{-1} m^{-2} s^{-1} up to 1000 µmol^{-1} m^{-2} s^{-1}, followed by 200 µmol steps up to 2000 µmol^{-1} m^{-2} s^{-1}. Rates of respiration (r) were always measured as O_2 consumption in the dark immediately after the light response. This yielded a photosynthesis-irradiance curve from which

the optimal light intensity for net photosynthesis was estimated using a modification of Eilers' photoinhibition model (Eilers & Peeters 1988) fitted via non-linear least squares regression (Fig. S2):

$$np(I) = \frac{np_{max}I}{(np_{max}/\alpha I_{opt}^{2})\,I^{2} + \left(1 - \left(\frac{2np_{max}}{\alpha I_{opt}}\right)\right)I + \frac{np_{max}}{\alpha}} - r \quad \text{(S3)}$$

where $np(I)$, is the rate of net photosynthesis at irradiance, I, np_{max} is the photosynthetic maximum that occurs at optimal light, I_{opt}, α controls the gradient of the initial slope and r is respiration. The optimum light intensity (I_{opt}, μmol^{-1} m^{-2} s^{-1}) for each taxon was then used for measuring net photosynthesis at all other assay temperatures in the acute thermal gradient experiments. This makes the assumption that I_{opt} does not vary with instantaneous temperature (Schaum *et al.* 2017). Instantaneous rates of net photosynthesis (at I_{opt}) and respiration were then taken at temperatures ranging from 5 to 50 ºC. Rates of gross photosynthesis were calculated by summing rates of net photosynthesis and respiration.

Rates of photosynthesis and respiration were normalised to biomass by expressing each rate measurement per unit of chlorophyll a. We used chlorophyll a as our biomass proxy as changes in the amount of chlorophyll a directly reflect changes in the photosynthetic capacity of organisms and ecosystems. Chlorophyll a extraction was achieved for each incubation by grinding the sample with methanol until all tissue had been broken down, centrifugation and measuring chlorophyll a extinction coefficients on a spectrophotometer. Total chlorophyll a (μg) was then calculated by measuring absorbance at 750 nm, 665 nm and 632 nm.

$$\text{Chl } a = (13.26(A_{665} - A_{750}) - 2.68(A_{665} - A_{750})) \times 10^{-3} \quad \text{(S4)}$$

Acute temperature responses of chlorophyll-normalised gross and net photosynthesis and respiration were fitted to the modified Sharpe-Schoolfield equation for high temperature inactivation (Equation 1).

Measuring *in situ* rates of ecosystem-level gross primary production

Ecosystem metabolism was calculated from measurements of dissolved oxygen over time in each stream using the single station method (Odum 1956). Sensors were deployed in all streams and at multiple sites within a stream where temperature gradients existed within streams due to differential geothermal warming. Dissolved oxygen concentration and temperature were monitored at 1-minute intervals using miniDOT optical dissolved oxygen loggers (PME Inc) (Fig. S3 & Fig. S5). Light sensors **(**Licor LI-193 spherical quantum sensor**)** were deployed simultaneously at two sites in the centre of the catchment. Physical variables of each stream, including the depth (m), width (m), velocity (m s^{-1}, measured using Hatch FH950), were measured along horizontal transects at approximately 10 m intervals up to the source of the stream. Values for depth, width and velocity were averaged across the reach (Table S4).

The change in O_2 concentration at a single station between two subsequent measurements (ΔDO) can be approximated as:

$$\Delta DO = \frac{[O_2]_t - [O_2]_{t-1}}{\Delta t} \quad \text{(S5)}$$

with $[O_2]_t$ the concentration of oxygen (mg L^{-1}) at time *t* and can be modelled using a framework based on the Odum's O_2 change technique (Odum 1956):

$$\Delta DO = GPP - ER \pm G \quad \text{(S6)}$$

where ΔDO is the composite of volumetric gross primary productivity, GPP (g m^{-3} min^{-1}), minus volumetric ecosystem respiration, ER (g m^{-3} min^{-1}) and G is the net

exchange of oxygen with the atmosphere (g O_2 m^{-3}). The net exchange of oxygen with the atmosphere (G) is the product of the O_2 gas transfer velocity, K (m min^{-1}), and the O_2 concentration gradient between the water body and the atmosphere (temperature and atmosphere corrected DO concentration at 100% saturation minus $[O_2]_t$) over the measurement interval.

The gas transfer velocity, K, was calculated using the surface-renewal model and corrected for the stream temperature:

$$K = 50.8\,V^{0.67} \times D^{-0.85} \times 1.024^{(T-20)} \quad \text{(S7)}$$

where V is velocity (cm s^{-1}), D is the mean stream depth (cm) adjusted for stream temperature, T (Bott 1996). This equation returns K in cm hr^{-1} which was subsequently converted into m min^{-1}. Estimated rates of reaeration, derived using the surface renewal model from measurements of velocity and depth, correspond well to reaeration rates measured experimentally using propane additions in an adjacent Icelandic catchment with comparable physico-chemical characteristics (see Fig. S8; Demars *et al.* 2011).

Measurements of dissolved oxygen concentration, light and temperature were averaged over 15 minute intervals for each 24-hour period. The net metabolic flux for a given measurement interval is equal to $\Delta\,DO - G$. During the night (where light < 5 µmol m^{-2} s^{-1}), GPP is zero, so the net metabolic flux is equal to ER. During the day, ER was determined by interpolating average ER over the defined night period. GPP for each daytime interval was the difference between net metabolism flux and interpolated ER. Daily volumetric rates of GPP (g O_2 m^{-3} day^{-1}) were calculated as the sum of the 15-minute rates over each 24-hour period. Volumetric rates were converted to areal units (g O_2 m^{-2} day^{-1}) by multiplying by the mean water depth of

the stream reach. At the end of the two years of sampling, we had 39 daily estimates of GPP across the 15 sites (Table S3).

In 2016, we also measured autotrophic biomass density (g Chl a m^{-2}) across the catchment by taking measurements of chlorophyll a. Autotrophic biomass was estimated by removing all organic material from a 30 cm^2 template on 3 randomly chosen rocks from each stream. Biofilm and plant material was removed from within the sample area using forceps and a stiff bristled brush, rinsed with distilled water and the slurry decanted into a Falcon tube. Chlorophyll was then extracted and quantified using the protocol detailed above. The total autotrophic biomass, M_s, of each stream reach was estimated by multiplying average autotrophic biomass density by the total reach area, which was estimated from the mean width and the upstream distance the oxygen sensor integrated over (Chapra & Di Toro 1991; Demars *et al.* 2015),

$$d = \frac{3V \times depth}{K} \qquad \text{(S8)}$$

where three times the velocity of the stream (V, m min^{-1}), multiplied by stream depth (m) and divided by the gas transfer velocity (K; m min^{-1}) gives the approximation of the distance upstream integrated by the single station method (d; m) (Grace & Imberger 2006). Biomass-corrected rates of GPP per stream (g O_2 g Chl a^{-1} day^{-1}) were calculated by dividing areal rates of GPP by the total autotrophic biomass, M_s, in the upstream reach.

Comparison of measured and modelled reaeration rates

To assess the robustness of our modelled values of reaeration, we compared measurements of the reaeration rate made in nearby streams in Iceland with comparable physical characteristics using propane additions (from Demars *et al.* 2011), to values estimated using the surface renewal model (eq. 14, main text). In

Demars *et al.* (2011), the reaeration rate was measured using a tracer study, where propane was bubbled continuously across the width of the stream at an upstream station. Water samples were taken at a downstream station and analysed by gas chromatography back in the laboratory (see for a more detailed description of the methods). The change in propane concentration the over the reach and the travel time were used to estimate the reaaeration rate, K (min^{-1}).

We compared the measured values of reaeration, K (min^{-1}), from Demars *et al.* (2011) to estimated values of K derived Eq. 14 (main text) and measurements of velocity, depth and temperature for those streams. We found a strong correlation between modelled and measured values of K with 95% confidence intervals on the slope that included unity (slope = 1.13, 95% CI: 0.76 – 1.50) and an R^2 = 0.61 (Fig. S8). In addition, we examined potential biases by plotting the residuals of the ln-ln plot of modelled vs measured reaeration against stream temperature (see Fig. S9). This analysis demonstrates that the model residuals do not vary systematically with stream temperature. Consequently, we are confident that estimates of reaeration derived from the surface renewal model are robust for the streams included in our survey.

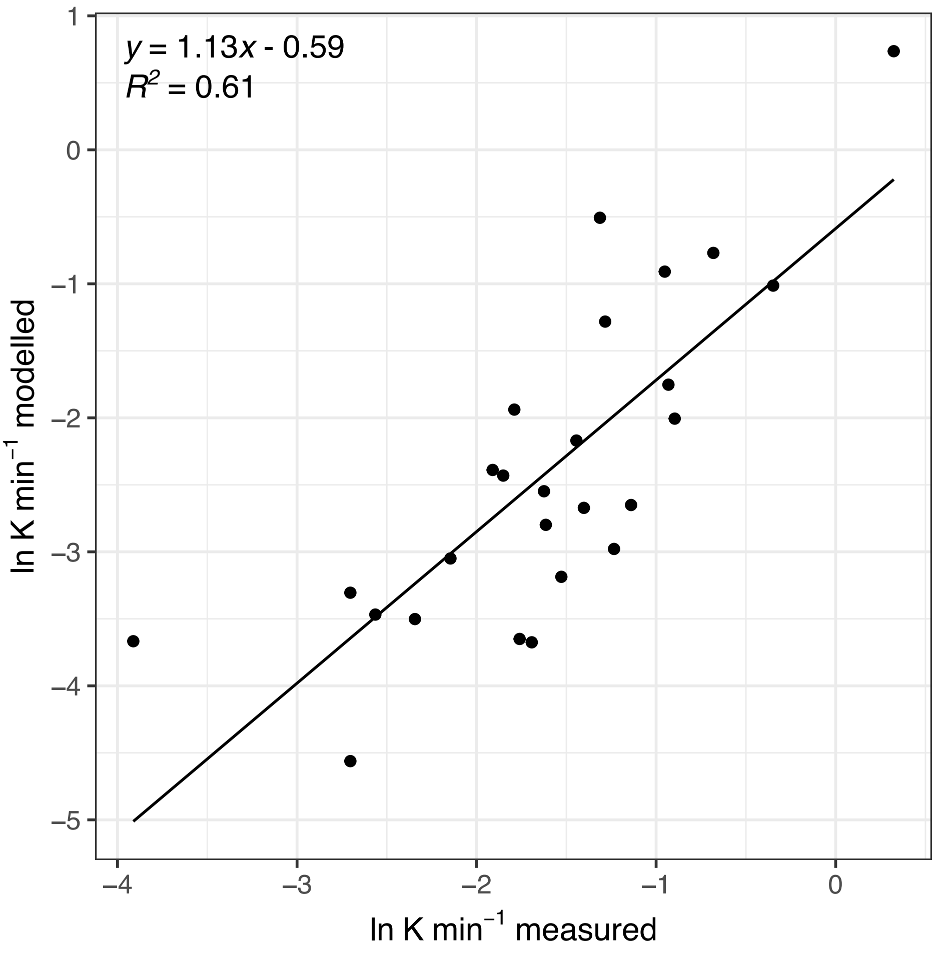

Figure S8. Comparison of modelled and measured rates of reaeration. Rates of measured reaeration using a propane tracer study are positively correlated with those derived using the surface renewal model (eq. 14; main text) with slope that was statistically indistinguishable from unity.

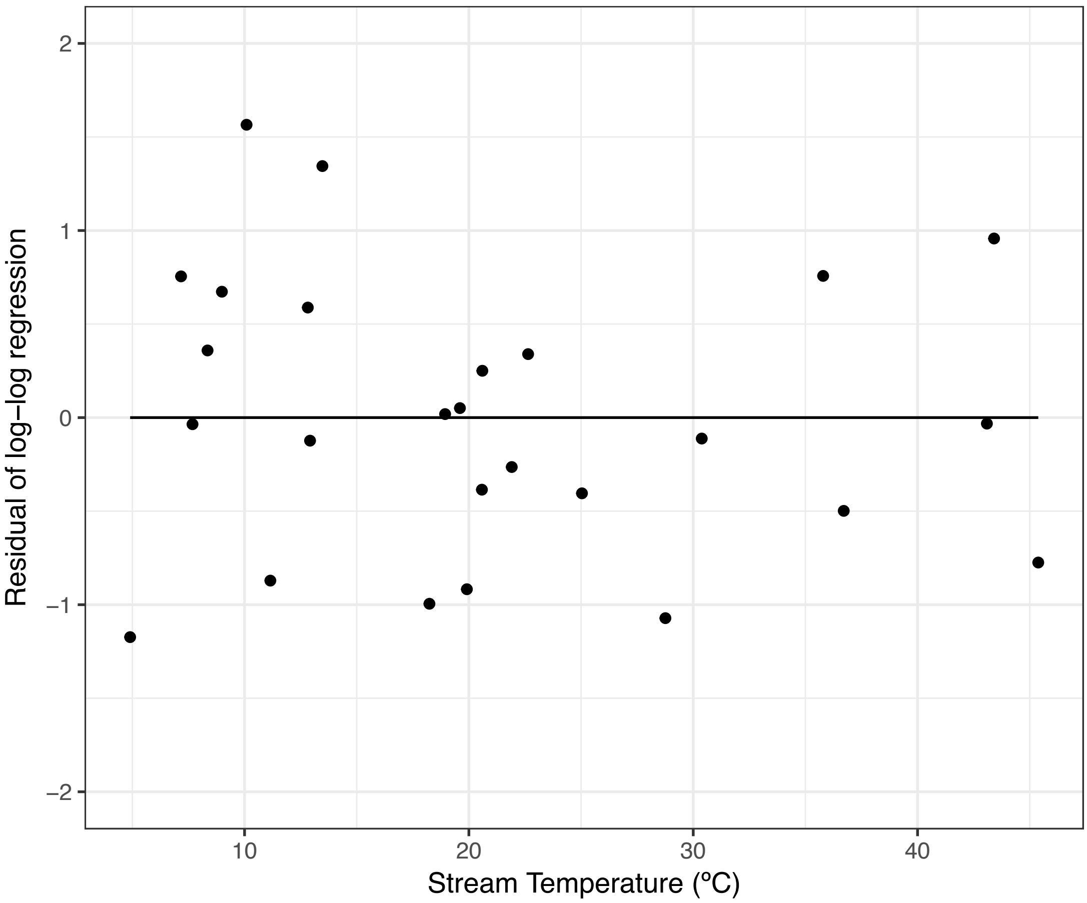

Figure S9. The relationship between the residuals of modelled *vs* measured reaeration with stream temperature. There was no systematic relationship between the residuals of the modelled vs. measured reaeration rates suggesting uncertainties in the modelled estimates of reaeration are unlikely to bias the observed temperature dependence of gross primary production.

References

1.Bott, T. (1996). Primary productivity and community respiration. In: *Methods stream Ecol. Elsevier Acad. Press*. San Diego

2.Chapra, S. & Di Toro, D. (1991). Delta method for estimating primary production, respiration, and reaeration in streams. *J. Environ. Eng.*, 117, 640–655

3.Demars, B.O.L., Russell Manson, J., Ólafsson, J.S., Gíslason, G.M., Gudmundsdóttir, R., Woodward, G., *et al.* (2011). Temperature and the metabolic balance of streams. *Freshw. Biol.*, 56, 1106–1121

4.Demars, B.O.L., Thompson, J. & Manson, J.R. (2015). Stream metabolism and the open diel oxygen method: Principles, practice, and perspectives. *Limnol. Oceanogr. Methods*, 13, 356–374

5.Eilers, P.H.. & Peeters, J.C.. (1988). A model for the relationship between light intensity and the rate of photosynthesis in phytoplankton. *Ecol. Modell.*, 42, 199–215

6.Grace, M.R. & Imberger, S.J. (2006). "Stream metabolism : Performing and interpreting measurements". *Water Studies Centre Monash University, Murray Darling Basin Commission and New South Wales Department of Environment and Climate Change* (2006).

7.Kirkwood, D. (1996). Nutrients: practical notes on their determination in seawater. *ICES Tech. Mar. Environ. Sci.*

8.Odum, H.T. (1956). Primary production in flowing waters. *Limnol. Oceanogr.*, 1, 102–117

9.Schaum, C.-E., Barton, S., Bestion, E., Buckling, A., Garcia-Carreras, B., Lopez, P., *et al.* (2017). Adaptation of phytoplankton to a decade of experimental warming linked to increased photosynthesis. *Nat. Ecol. Evol.*, 1, 94

10.Schoolfield, R.M., Sharpe, P.J. & Magnuson, C.E. (1981). Non-linear regression of biological temperature-dependent rate models based on absolute reaction-rate theory. *J. Theor. Biol.*, 88, 719–731

11.Yvon-Durocher, G., Allen, A.P., Bastviken, D., Conrad, R., Gudasz, C., St-Pierre, A., *et al.* (2014). Methane fluxes show consistent temperature dependence across microbial to ecosystem scales. *Nature*, 507, 488–491
