# Supplementary material for: Metabolic compensation constrains the temperature dependence of gross primary production
Source: Ecol Lett. 2017 Aug 29;20(10):1250–60. doi: 10.1111/ele.12820 (PMC6849571; doi:10.1111/ele.12820)
Supplement: Supplementary file 2 [file ELE-20-1250-s002.pdf]

Supplementary Information: Additional Authors

Much of the data underpinning this work was collected by students as part of an undergraduate field course to Iceland. The students who contributed to data collection are named below. Their current correspondence is:

Centre for Ecology and Conservation, College of Life and Environmental Sciences, University of Exeter, Penryn, Cornwall, TR10 9FE, U.K.

Student Research Team

Hannah O'Sullivan,
Lily Chambers,
Nicola Vallance-Crabb,
Emily Carter,
Emma Holden,
Ellinor Alseth,
Amy Cole,
Rosanna Kettlewell,
Conor Watson,
Joe Parker,
Ben Lawton,
Piers Roberts,
Abigail Lloyd,
Tabitha Sveinsson,
Maurice Kot,
Honor-May Moggridge,
Tia McFarlane,
James O'Mahony,
Jake Rogers,
Hugh Balmer,
Oscar Lozada,
Molly Meadows,
Harriet Lavender,
Abigail Parker,
Jessica Haines,
Meike Simms,
Dominic James,
Elsa Spoors,
Flora Rendell-Bhatti,
Alex Marshall,
Meriel Anderson,
David Williamson,
Hannah Emes,
Adam Hughes,
Jacca Deeble,
Anthony Coleman,
Ben Jenkinson,
Sarah Blake
